# The Distribution and ‘*In Vivo*’ Phase Variation Status of Haemoglobin Receptors in Invasive Meningococcal Serogroup B Disease: Genotypic and Phenotypic Analysis

**DOI:** 10.1371/journal.pone.0076932

**Published:** 2013-09-30

**Authors:** Jay Lucidarme, Jamie Findlow, Hannah Chan, Ian M. Feavers, Stephen J. Gray, Edward B. Kaczmarski, Julian Parkhill, Xilian Bai, Ray Borrow, Christopher D. Bayliss

**Affiliations:** Public Health England, Manchester, United Kingdom; National Institute of Biological Standards and Control, Potters Bar, United Kingdom; The Wellcome Trust Sanger Institute, Cambridge, United Kingdom; University of Leicester, Leicester, United Kingdom; Cornell University, United States of America

## Abstract

Two haemoglobin-binding proteins, HmbR and HpuAB, contribute to iron acquisition by *Neisseria meningitidis*. These receptors are subject to high frequency, reversible switches in gene expression - phase variation (PV) - due to mutations in homopolymeric (poly-G) repeats present in the open reading frame. The distribution and PV state of these receptors was assessed for a representative collection of isolates from invasive meningococcal disease patients of England, Wales and Northern Ireland. Most of the major clonal complexes had only the HmbR receptor whilst the recently expanding ST-275-centred cluster of the ST-269 clonal complex had both receptors. At least one of the receptors was in an ‘ON’ configuration in 76.3% of the isolates, a finding that was largely consistent with phenotypic analyses. As PV status may change during isolation and culture of meningococci, a PCR-based protocol was utilised to confirm the expression status of the receptors within contemporaneously acquired clinical specimens (blood/cerebrospinal fluid) from the respective patients. The expression state was confirmed for all isolate/specimen pairs with <15 tract repeats indicating that the PV status of these receptors is stable during isolation. This study therefore establishes a protocol for determining *in vivo* PV status to aid in determining the contributions of phase variable genes to invasive meningococcal disease. Furthermore, the results of the study support a putative but non-essential role of the meningococcal haemoglobin receptors as virulence factors whilst further highlighting their vaccine candidacy.

## Introduction

*Neisseria meningitidis* (the meningococcus) is a commensal of the human nasopharynx with an overall prevalence of approximately 10% [1]. It is a leading cause of meningitis and septicaemia globally and is associated with significant morbidity and mortality [2]. There are five main capsular serogroups associated with invasive meningococcal disease (IMD; serogroups A, B, C, W and Y) of which serogroups A, C, W and Y are preventable by means of efficacious glycoconjugate vaccines [3]. The polysaccharide of serogroup B meningococci (MenB), a leading cause of IMD in industrialized nations, is poorly immunogenic in humans [4]. MenB vaccine development has largely, therefore, focused upon sub-capsular antigens [2], [5]–[9]. Efforts to broaden coverage are, however, ongoing.

Within the human host, the essential nutrient iron is mainly sequestered within cells. Extracellular iron tends to be complexed with various proteins including transferrin, lactoferrin, haemoglobin (Hb) and Hb-haptoglobin, the respective availabilities of which differ according to anatomical site. For example, transferrin is mostly located in the serum, whilst lactoferrin is found in secretions [10]. Meningococci are able to acquire iron from these complexes via a variety of surface receptors [10]. As addition of free iron or transferrin is required to establish IMD in mouse models of infection [11], these receptors constitute potential virulence factors. The surface expression of the receptors also confers vaccine candidacy.

The TonB dependent HmbR and HpuAB systems are each able to bind Hb, whilst the latter also binds the Hb-haptoglobin complex [12], [13]. HmbR is an 89 kDa transmembrane protein, whilst HpuAB comprises a 37 kDa lipoprotein (HpuA) and an 85 kDa transmembrane protein (HpuB). Both systems undergo phase variation (PV) owing to homopolymeric (poly-G) tracts in the open reading frames of *hmbR* and *hpuA* [14]. These receptors are variably distributed among meningococci, with an overrepresentation of *hmbR,* and underrepresentation of the *hpuAB*-only genotype among IMD isolates [15], [16]. Notably, it has been demonstrated that the majority of isolates belonging to clonal complexes (CCs) with relatively high disease to carriage ratios possessed both receptor systems [16].

Analysis of PV status in two collections of isolates indicated that 91% of IMD isolates possessed one or both receptors in the PV ON configuration compared with only 71% among carriage isolates [16]. Both HmbR and HpuA (the surface exposed component of HpuAB) exhibit significant levels of amino acid sequence variation [16], [17]. Furthermore, the putative expression and immunogenicity of *hmbR* during IMD has been demonstrated by the presence of anti-HmbR antibodies in convalescent sera [10]. Collectively these findings suggest that the expression of at least one Hb receptor is important for IMD and that both receptors are subject to immune selective pressures, providing support for their potential role as both virulence factors and vaccine candidates.

In addition to *hmbR* and *hpuA*, meningococcal genomes contain multiple other phase variable genes, with homopolymeric tracts being a major mechanism for switching gene expression ON and OFF [18]. There have been few attempts to determine the PV status of these genes in IMD isolates even though several of them encode factors likely to affect survival of meningococci in systemic sites [18]. The study of the Hb receptors was one of the first to link a particular expression state with the disease state [16]. This and previous studies were, however, performed on isolates that had typically undergone several passages on blood-containing media following initial isolation, thus raising the possibility of *in vitro* selection of a PV state unrepresentative of that which prevails during IMD.

In this report we describe a methodology for direct analysis of the PV status of meningococcal genes in clinical specimens. This approach removes the potential bias associated with culture of meningococcal strains and is applicable to all genes whose PV is mediated by simple sequence repeat tracts. In addition, the study assessed the distribution and PV status of *hmbR* and *hpuA* among recent representative IMD cases from England, Wales and Northern Ireland in order to further explore their potential role as virulence factors and vaccine candidates.

## Methods

### Ethical approval

Favorable ethical approval for the study was provided by the National Research Ethics Service (research ethics committee reference number 11/NE/0235). The requirement for consent was waivered by the ethical review board as the study used anonymised samples that had already been collected for clinical need.

### Isolates and clinical specimens

The study samples comprised 80 English, Welsh and Northern Irish MenB IMD isolates (received by the Health Protection Agency Meningococcal Reference Unit (MRU) between December 2008 and April 2011) and their corresponding clinical specimens (cerebrospinal fluid (CSF; n = 11) or blood or derivatives thereof (n = 69)). The isolates were selected to represent (in terms of CC) the diversity and distribution of recent English, Welsh and Northern Irish MenB IMD isolates as determined for the epidemiological year 2007/8 [19]. The CC distribution of the selected isolates is presented in table 1. Isolate/specimen pairs with non-culture real-time PCR *ctrA* cycle thresholds >35 [20] were excluded from the study in order to maximise the ability to detect the Hb receptor genes.

**Table 1:** Comparison of the clonal complex distribution of the study isolates and all English, Welsh and Northern Irish serogroup B invasive meningococcal disease isolates for the epidemiological year 2007/8.

Clonal complex	Proportion of isolates (number)	
	Study (80)	2007/8 (539)
11	2.5% (2)	1.1% (6)
18	1.3% (1)	1.7% (9)
32	7.5% (6)	5.9% (32)
35	1.3% (1)	1.5% (8)
60	3.8% (3)	2.0% (11)
103	1.3% (1)	0.2% (1)
162	5.0% (4)	1.9% (10)
213	6.3% (5)	9.5% (51)
269	27.5% (22)	33.0% (178)
461	2.5% (2)	2.2% (12)
1157	3.8% (3)	0.4% (2)
41/44	26.3% (21)	31.5% (170)
UA	11.3% (9)	7.2% (39)
other	n/a	1.9% (10)

Isolates used in the study were selected to represent the diversity and distribution (in terms of clonal complex) of all English, Welsh and Northern Irish invasive MenB isolates from 2007/8. Note that a proportion of the predominant CCs (cc269 and cc41/44) were forfeited to include lesser CCs. Other variations were due to the limited availability of multilocus sequence typed isolates with accessible clinical specimens. UA =  unassigned STs. n/a =  not applicable.

### DNA extraction

DNA was extracted using the Qiagen DNeasy blood and tissue kit (Qiagen, Crawley, United Kingdom). Isolates were cultured overnight on Columbia agar plus 5% horse blood prior to DNA extraction as previously described [21]. DNA was extracted from a broad sweep of multiple colonies to avoid selection of unrepresentative phase variants. Extractions from clinical specimens were performed in accordance with the manufacturer's DNeasy Blood & Tissue Handbook (Qiagen, 2006 edition).

### Molecular analyses for detection of *hmbR* and *hpuA* and characterization of the homopolymeric tracts and closely flanking regions

Primers used in the study, and PCR/sequence analysis conditions can be viewed in table 2. A schematic diagram of the workflow for molecular analyses is provided in figure S1. Sequence and fragment analyses were performed on a 3130xL Genetic Analyzer installed with a 50 cm capillary array (Life Technologies Ltd, Paisley, United Kingdom).

**Table 2:** Primers used for PCR and sequence and fragment analyses for *hmbR* and *hpuA*.

Target	PCR/Seq/frag^a^	Primer ID (direction)	Primer sequence (5′ to 3′)	Reference	Approx size (bases)
*hmbR*	PCR/Seq/frag^b^	*hmbR-RF3* (fwd)	TGCCAACCTCTTTTACGAATGG	[25]	400
		*hmbR-RF4* (rev)	GCTACTGAACACGTCGTTCC	[35]	
	PCR^c^	*hmbRzF* (fwd)	CCACA(A/G)CTT(C/T)TTGGGTAAGATTGC	This study	1000
		*hmbRzR* (rev)	GACGCTACTTTGTCCACATTCAGACG	This study	
	PCR^d^	*hmbReF* (fwd)	AAAT(C/T)AACGA(C/T)AACCACCGCATCG	This study	600
		*hmbRyR* (rev)	GGCATTCAATTCCTGAGGCGTCA	This study	
	PCR^e^	*exl3-seqF* (fwd)	GGCGGAGTGCAAAATGATGC	[15]	600
		*exl3-seqR* (rev)	GCCATCTTTTAATTTAGCCGC	[15]	
*hpuA*	PCR/Seq/frag^b^	*hpuAC* (fwd)	ATGCGATGAAATACAAAGCCC	[25]	350
		*hpuA350Rev* (rev)	GGATGAAAGGGCGTATTGCGC	[25]	
	PCR^f^	*hpuAFnest2* (fwd)	CAAATCCGCCAACGAAGCGAT(C/T)AA	This study	2000
		*P26.85* (rev)	GGGAAACGCTTGGGCGATGG	[36]	
	PCR^g^	*Hpu-pmt* (fwd)	CCGATTTTTGCACCGACCCAC	This study	600
		*hpuR-Seq3* (rev)	GAGGTCGATTTCGCCGTTGG	This study	
	PCR^h^	*hpuA-for1* (fwd)	GCAACAATGCCTTGTCATCC	[16]	1000–3000
		*hpuA-rev13* (rev)	TGATCGAAATGGGCGTACTC	[16]	

a Purpose; conditions

b Characterisation of homopolymeric tract and closely flanking regions; PCR - [MgCl_2_] = 2.5 mM, 25 (up to 45 for direct non-culture fragment analysis) cycles of (95°C – 30 seconds, 53°C – 30 seconds, 72°C – 60 seconds). Sequence analysis annealing temperature  = 53°C. For fragment analysis, primers *hmbR-RF3* and *hpuA350Rev* were FAM-labeled.

c ‘Round 1’ nested PCR for non-culture characterization of homopolymeric tract and closely flanking regions; [MgCl_2_] = 3 mM, 45 cycles of (95°C – 30 seconds, 56°C – 30 seconds, 72°C – 60 seconds).

d‘Round 2’ nested PCR for non-culture characterization of homopolymeric tract and closely flanking regions; [MgCl_2_] = 3 mM, 25 cycles of (95°C – 30 seconds, 56°C – 30 seconds, 72°C – 60 seconds).

eConfirmation of presence of *exl3* (that replaces *hmbR*); [MgCl_2_] = 3 mM, 35 cycles of (95°C for 30 seconds, 56°C for 30 seconds, 72°C for 60 seconds).

f’Round 1’ nested PCR for non-culture characterization of homopolymeric tract and closely flanking regions; [MgCl_2_] = 3 mM, 45 cycles of (95°C – 30 seconds, 57°C – 30 seconds, 72°C – 150 seconds).

g‘Round 2’ nested PCR for non-culture characterization of homopolymeric tract and closely flanking regions; [MgCl_2_] = 3 mM, 25 cycles of (95°C – 30 seconds, 59°C – 30 seconds, 72°C – 60 seconds).

h Confirmation of absence of *hpuA*; [MgCl_2_] = 3 mM, 35 cycles (95°C – 30 seconds, 53°C – 30 seconds, 72°C – 180 seconds).

Seq =  sequence analysis

Frag =  fragment analysis

### Detection of *hmbR* and *hpuA* genes

The presence/absence of *hmbR* and *hpuA* among cultures was determined in accordance with Tauseef *et al*. [16]. Briefly, PCR and sequence analyses were performed using proximal, intragenically targeted primers (*hmbR-RF3* and *hmbr-RF4* (*hmbR*) and *hpuAC* and *hpuA350Rev* (*hpuA*)), closely flanking the respective homopolymeric tracts. For non-culture clinical specimens, sequence analysis (as above) was preceded by a nested PCR in which the initial PCR (‘round 1’, using extracted DNA as template) used remote intragenically targeted primers (*hmbRzF* and *hmbRzR* (*hmbR*), or *hpuAFnest2* and *P26.85* (*hpuA*)), and the second PCR (‘round 2’, using round 1 PCR product as template) used intermediate primers (*hmbReF* and *hmbRyr* (*hmbR*) or *Hpu-pmt* and *hpuR-Seq3* (*hpuA*)).

The absence of *hmbR* was confirmed by PCR in which intragenically targeted primers (*exl3-seqF* and *exl3-seqR*) were used to detect the *exl3* gene that replaces *hmbR* in the respective isolates, in accordance with Harrison *et al*. [15]. The absence of *hpuA* was confirmed by PCR amplification of the corresponding locus using extragenically targeted primers (*hpuA-for1* and *hpuA-rev13*), in accordance with Tauseef *et al*. [16].

### Characterization of homopolymeric tracts and flanking regions

Homopolymeric tracts and flanking sequences were characterised by a combination of sequence analysis (above) and fragment analysis of corresponding FAM labeled PCR products (amplified using primers *hmbR-RF3*-FAM and *hmbr-RF4* (*hmbR*), and *hpuAC* and *hpuA350Rev-*FAM (*hpuA*)) against a GeneScan 500 LIZ size standard (Life Technologies Ltd) [16]. Non-culture fragment analysis of clinical specimens initially made use of a nested approach incorporating the respective round 1 PCRs (above). In order to reduce putative strand slippage artefacts generated for longer homopolymeric tracts, the total number of PCR cycles (45 plus 25 cycles for the nested protocol) was reduced by using a direct, non-nested protocol in conjunction with 25 to 45 cycle repeats (adjusted to maintain sensitivity while minimizing the number of cycles). The sensitivity of the non-nested, non-culture fragment analysis was further enhanced, where necessary, by increasing the pre-electrophoresis sample injection time on the sequence analyser (from a standard time of 6 seconds to between 18 and 50 seconds) and/or ethanol/sodium acetate precipitation of FAM-labeled PCR products that were then dissolved in formamide prior to electrophoresis.

Fragment sizes were determined using Peak Scanner software (v1.0, Life Technologies Ltd). These were compared with the corresponding sequence data in order to associate corresponding homopolymeric tract lengths (figure S2 (a) and (b)). Where multiple fragment peaks (differing in the number of homopolymeric tract repeats) were obtained, the relative amounts of the respective primary (1°) and secondary (2°) products were estimated by calculating the ratio of the corresponding peak areas (obtained using the Peak Scanner software; figure S2 (c)).

In order to exclude the potential effect of non-PV mutations on expression status, full length allele sequences were obtained from whole genome sequence data obtained using Illumina sequence technology (Illumina, California, USA).

### Phenotypic analyses

The ability to utilise Hb (and therefore the existence of at least one haemoglobin receptor in the ON state) was investigated among the isolates in accordance with Tauseef *et al*. [16]. Meningococcal suspensions (10–15 µL at optical density (650 nm) = 0.2) were spread-plated onto Mueller Hinton (MH) agar containing 40 µM desferal and incubated overnight (37°C with 5% CO2) in the presence of Hb impregnated discs (10 µL at 10 mg mL-1). Sterile water and transferrin (10 µL at 50 mg mL-1) and/or ferric nitrate (10 µL at 16.2 mg mL-1) impregnated discs and parallel cultures on plain Mueller Hinton agar (no desferal or nutritional supplements) served as controls. Nutritional supplements were obtained from Sigma-Aldrich (Dorset, UK).

## Results

### Distribution of *hmbR* and *hpuA*

**Figure 1:**
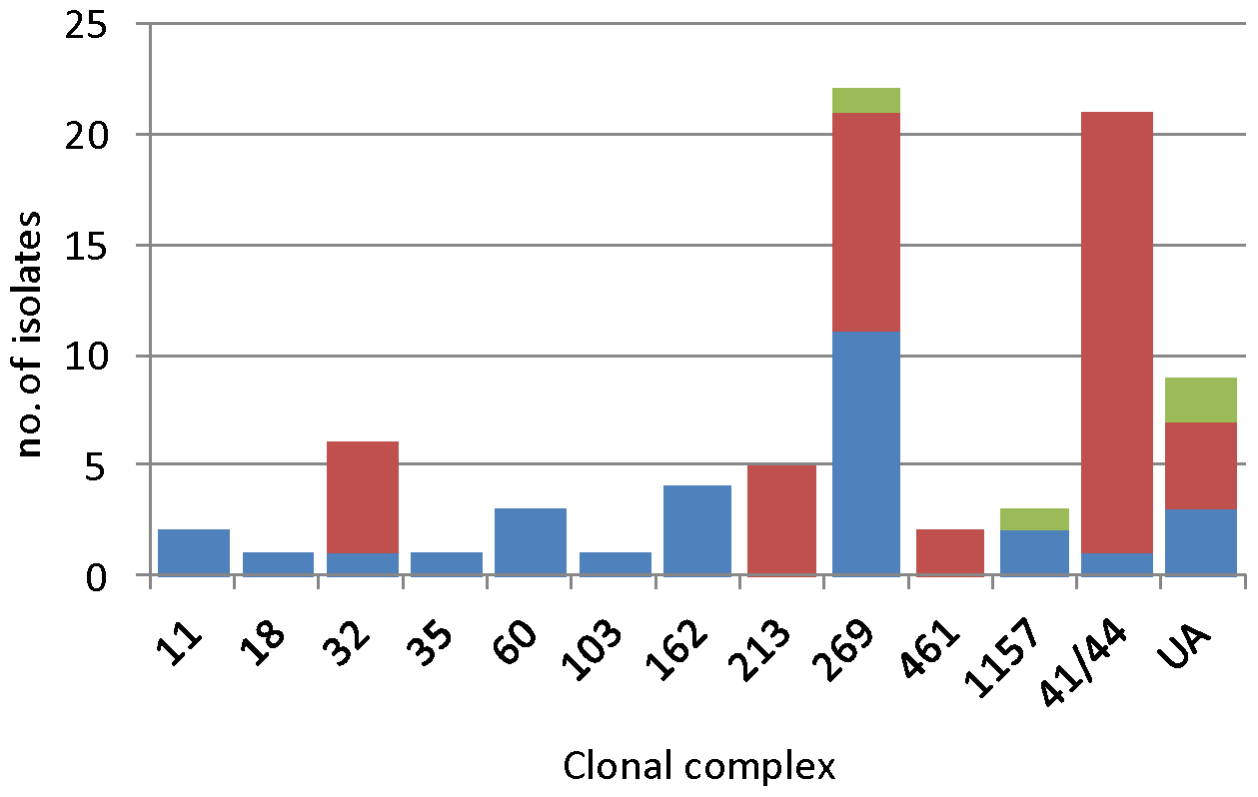
Clonal complex distribution of the combinations of *hmbR* and *hpuA* genes among a representative panel of English, Welsh and Northern Irish invasive serogroup B meningococcal isolates collected from 2008–2011. Blue bars indicate *hmbR:hpuA*, red bars indicate *hmbR*-only and green bars indicate *hpuA*-only. UA =  unassigned STs.

All of the 80 isolates possessed genes for at least one of the Hb receptors (figure 1). Among these, 57.5% possessed *hmbR* only, 5.0% possessed *hpuA* only, and 37.5% possessed both genes (designated *hmbR:hpuA*). The majority of isolates belonging to less prevalent CCs (<5 isolates) possessed both genes. Three of the most prevalent CCs, cc41/44, cc213 and cc32, comprised predominantly ‘*hmbR*-only’ isolates (95.2%, 83% and 100%, respectively). Contrastingly, the ST-269 clonal complex (cc269) was split between isolates comprising an *hmbR*-only (45.5%; all centered around sequence type (ST)-269 by eBURST analysis [19], table S1) or an *hmbR*:*hpuA* genotype (50%; of which 90.9% were centered around ST-275 [19]).

### Sequence analysis of homopolymeric tract regions of *hmbR* and *hpuA* among IMD isolates

Homopolymeric tracts are susceptible to strand slippage during PCR amplification and this susceptibility increases with tract length and the number of PCR cycles [22]. Affected PCR products comprise a heterogeneous population of amplicons with tract lengths varying around that of the template DNA. Upon sequence analysis this manifests itself as a series of superimposed, laterally shifted and progressively weakening chromatogram traces that occur immediately after the homopolymeric tract (in the direction of the respective chromatogram).

In the present study, dideoxy sequencing was performed on PCR products spanning the homopolymeric tracts and flanking regions. Among the isolates, alleles with >10 G repeats tended to yield the aforementioned heterogeneous products indicative of strand slippage during PCR and/or heterogeneous template DNA (and therefore a heterogeneous plate population). For tracts exceeding approximately 15 G repeats, the downstream sequence (with respect to the direction of the respective chromatogram) tended to be unreadable and could only be discerned from the antiparallel chromatogram.

**Figure 2:**
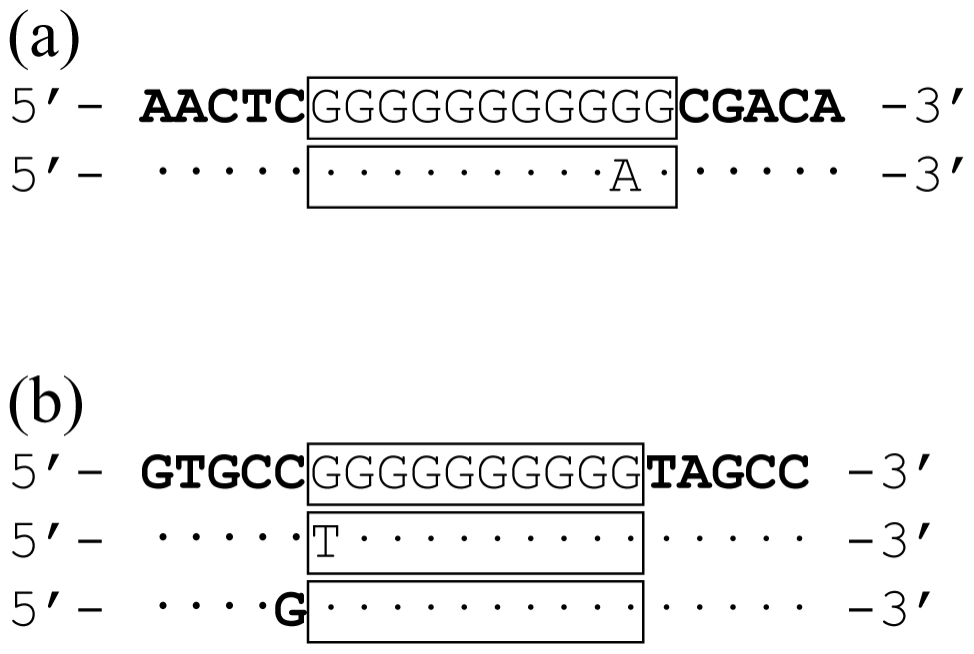
Sequence diversity in the homopolymeric tract regions of *hmbR* and *hpuA*. Due to polymorphisms in the homopolymeric tract and flanking regions, the phase variation expression status could not be reliably correlated with the absolute number of consecutive G repeats (absolute tract length), rather it corresponded with an ‘effective tract length’ between defined flanking regions. In the examples provided, tracts are illustrated as pairwise nucleotide alignments in which the defined flanking regions are highlighted using bold type whilst the effective tract length is denoted by a box. Pairwise nucleotide identities are denoted by dots. For *hmbR* (a) the defined flanking regions were AACTC and CGACA, respectively. In the upper example the effective and absolute tract lengths are the same (11 Gs vs 11 bases). The lower tract has a G to A substitution at the second to last G giving an absolute tract length of 9 Gs whilst the effective tract length remains 11 bases. For *hpuA* (b) the defined flanking regions were GTGC(C/G) and TAGCC, respectively. In the upper example the effective and absolute tract length are the same (10 Gs vs 10 bases). The middle example has a G to T substitution at the first G giving an absolute tract length of 9 Gs whilst the effective tract length remains 10 bases. The lower example has a C to G substitution immediately before the prototypical tract region giving an absolute tract length of 11 Gs whilst the effective tract length remains 10 bases.

Some inter-isolate sequence variation occurred within or adjacent to the repeat tracts. For example, one of the 76 *hmbR* sequences (obtained from isolate i4), contained a substitution within the homopolymeric tract in which the second to last G repeat was substituted for an A (figure 2a). Thus, whilst the absolute number of consecutive G repeats (‘absolute tract length’) was 9, the overall length of the corresponding region (‘effective tract length’; described by defined flanking regions on a pairwise nucleotide alignment; figure 2) was 11 bases. Similarly, nine of the 34 *hpuA* gene sequences had alterations in the sequence at the 5′ end of the repeat tract (examples of which are provided in figure 2b). One isolate (isolate i11) had a ‘G to T’ substitution thereby reducing the absolute tract length, whilst eight others (isolates i15, i17, i19, i59, i60, i61, i90 and i91) had a ‘C to G’ substitution thereby increasing the absolute tract length. In each case the effective tract length remained unaffected by the respective mutation. Since phase variation status is dependent on the overall length of the prototypical tract region, the effective tract length was utilised for predicting expression state in the subsequent analyses. Effective/absolute tract lengths can be viewed in table S1.

### Characterisation of homopolymeric tract lengths and PV status of *hmbR* and *hpuA* among IMD isolates

Owing to the heterogeneity of sequences containing relatively long homopolymeric tracts, the homopolymeric tract lengths of all 80 isolates were determined from a combination of sequence and fragment analyses.

**Figure 3:**
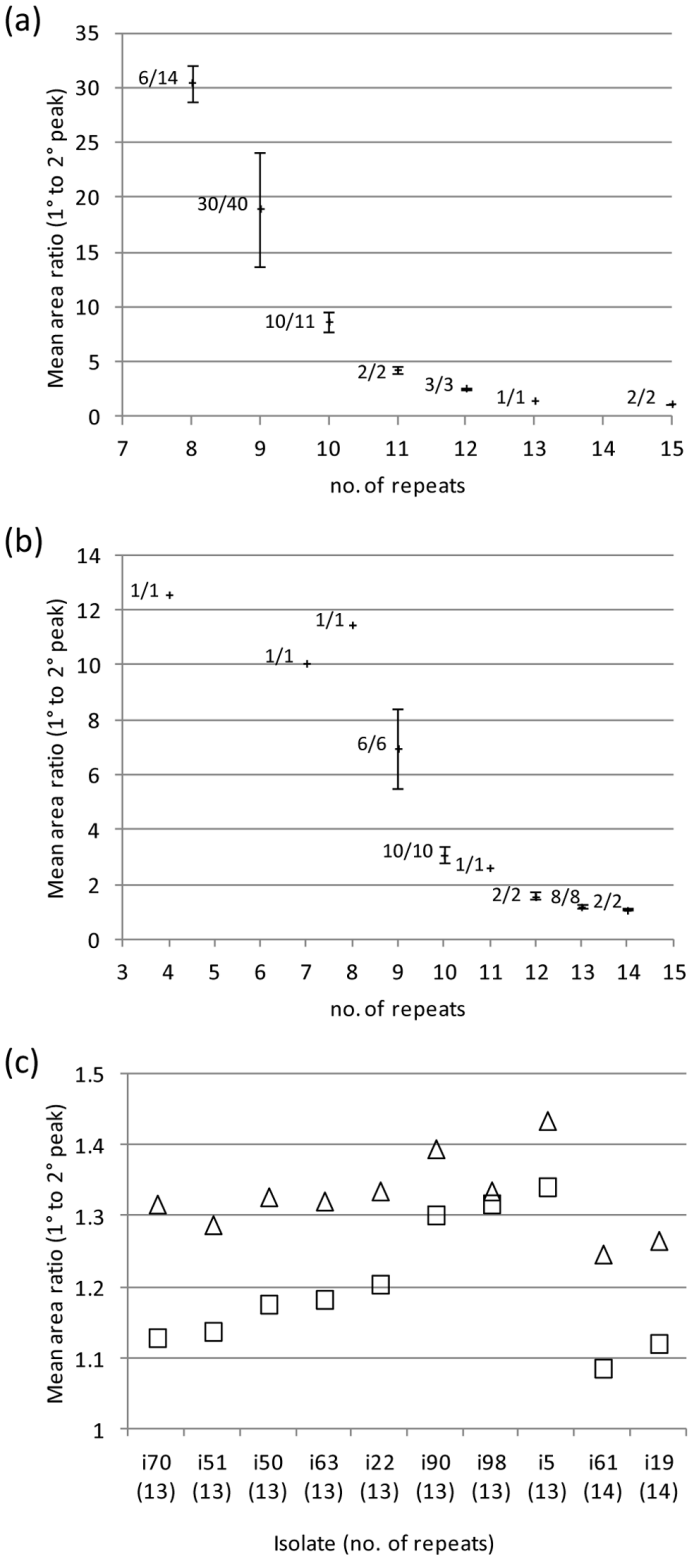
Influence of homopolymeric tract length on the heterogeneity of PCR products. For a proportion of the isolates, amplification across the homopolymeric repeat tracts of *hmbR* and *hpuA* generated multiple peaks on fragment analysis. The relative amounts of the respective primary (1°) and secondary (2°) products were estimated by calculating the ratio of the corresponding peak areas using Peak Scanner software (Life Technologies Ltd). Charts (a) and (b) illustrate a decrease in mean peak area ratio with increasing absolute homopolymeric tract length (corresponding to the 1° peak) for *hmbR* and *hpuA*, respectively. The proportions of isolates exhibiting heterogeneous fragments are indicated beside markers. Hairs indicate standard deviations. All PCRs utilised 25 cycle repeats. Chart (c) compares the mean peak area ratios obtained using 25 (squares) or 20 (triangles) PCR cycle repeats for *hpuA*+ isolates possessing 13 to 14 G repeats. In each case the mean peak area ratio increased when using fewer PCR cycle repeats.

Fragment analysis separates heterogeneous amplicon species according to length. The area below the corresponding fragment peaks provides an indication of the relative proportions of the respective amplicon species. In the present study the two peaks with the largest and second largest area were designated 1° and 2°, respectively (figure S2 (c)).The heterogeneity of the PCR products and the proportion of putatively artifactual fragments (as indicated by the ratio of the areas for the 1° and 2° peaks) increased as a function of tract length. For the default PCRs (25 cycle repeats), the mean 1° to 2° peak-area ratio for *hmbR* ranged from 30.4 for G8 tracts to 1.14 for G15 tracts (figure 3a). For *hpuA* the mean 1° to 2° peak area ratios ranged from 12.6 for G4 tracts to 1.10 for G14 tracts (figure 3b). A single isolate (isolate i15) possessed *hpuA* with a tract of approximately 19 G repeats by sequence analysis. On fragment analysis, this yielded >7 fragment peaks. The four largest peaks had area ratios (adjacent peaks) of 1.56, 1.07 and 2.02, respectively. The largest peak corresponded to a tract length of 16 G repeats which appeared incorrect when viewing the sequence analysis. To reduce the effect of strand slippage during PCR, *hpuA* fragment analysis was repeated for all isolates with ≥13 tract G repeats using a reduced number of PCR repeat cycles (from 25 to 20 cycles). The 1° peak was maintained for all isolates except isolate i15. In addition, the respective 1° to 2° peak-area ratios were seen to increase (figure 3c), thus supporting the initial results and predicted fragment sizes. The 1° peak of isolate i15 shifted to the right, however, suggesting that the original 1° peak was artifactual and that the isolate had an effective tract length of 18+ repeats which was more consistent with the chromatograms. The 1° to 2° peak area ratio for this isolate remained low (1.05), however, and so the exact number of repeats remained uncertain.

### Distribution of predicted effective tract lengths and genotypic PV status among isolates

**Figure 4:**
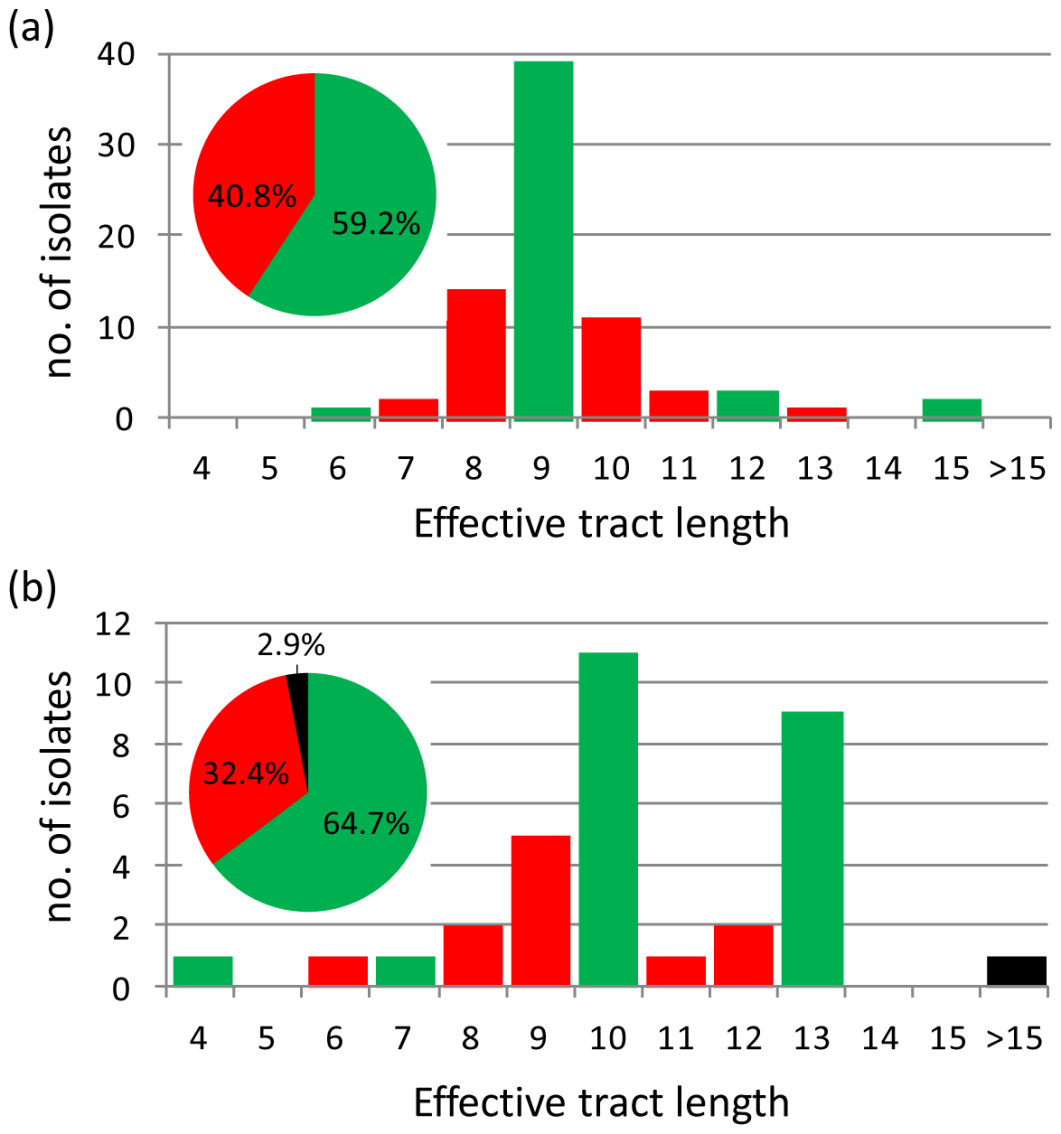
Distribution of effective homopolymeric tract lengths for *hmbR* and *hpuA* among a representative panel of English, Welsh and Northern Irish invasive serogroup B meningococcal isolates collected from 2008–2011. Bar charts show the number of isolates with a particular ‘effective homopolymeric tract length’ for *hmbR* (a) and *hpuA* (b). Green bars indicate a predicted ON expression state, red bars indicate a predicted OFF expression state, and black bars indicate an indeterminate expression state for a single isolate with a homopolymeric tract length of >15 G repeats. Inset are pie charts depicting the proportion of isolates representing each expression state for the respective genes.

The *hmbR* gene was considered ON for effective tract lengths divisible by 3, whilst *hpuA* was considered ON for effective tract lengths of 4, 7, 10 and so on (figure 4). Among the *hmbR*+ isolates, 59.2% of the tracts were in the ON configuration. The predominant effective tract length for this gene was 9 (51.3% overall) which accounted for 86.7% of effective *hmbR* tracts in the ON configuration. Among the *hpuA*+ isolates 64.7% of the effective tracts were in the ON configuration. The predominant effective tract lengths among these were 10 (11/34, 32.4% overall) and 13 (9/34, 26.5% overall), collectively accounting for 90.9% of effective *hpuA* tracts in the ON configuration.

**Figure 5:**
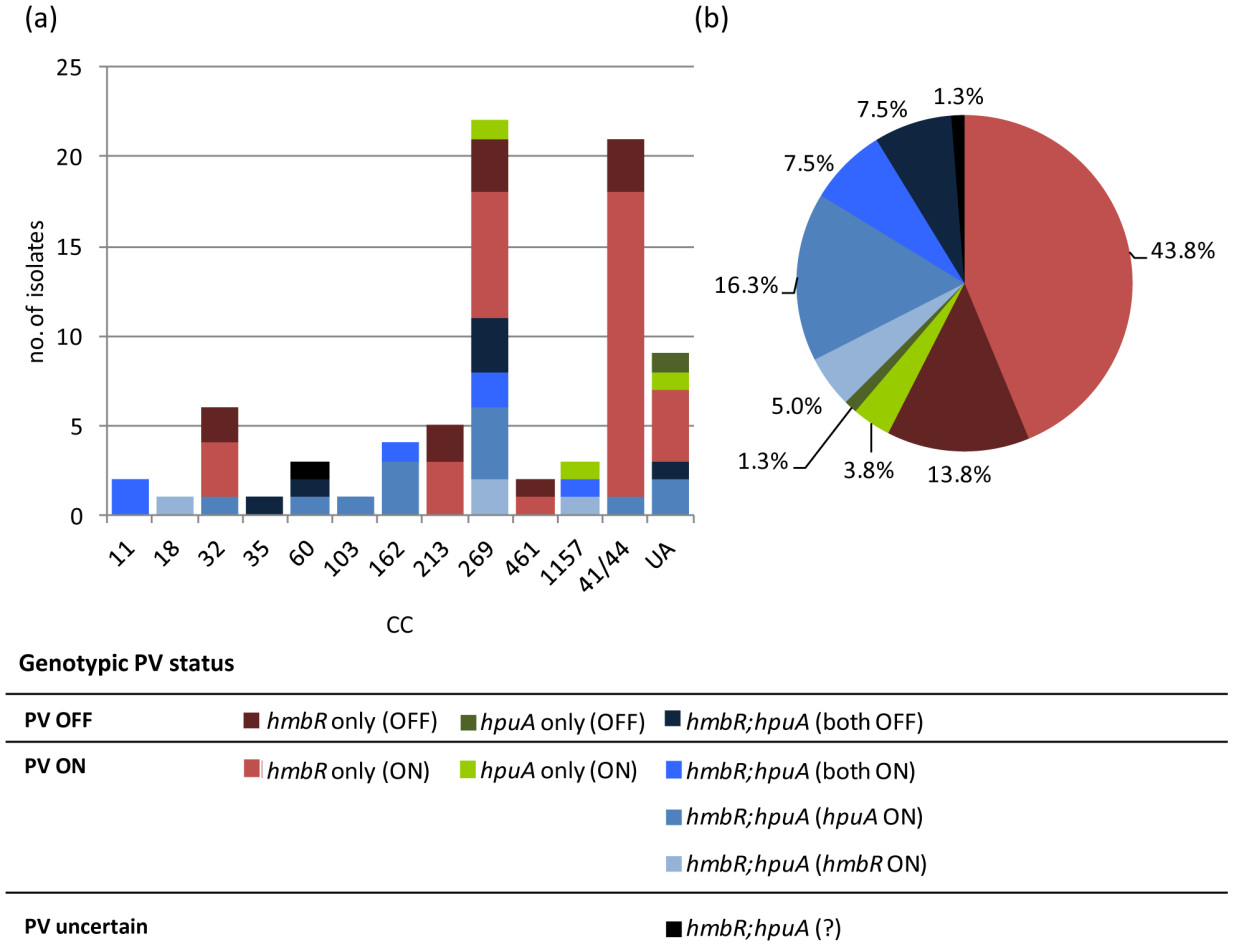
Distribution of genotypic phase variable expression status of *hmbR:hpuA* genotypes among a representative panel of English, Welsh and Northern Irish invasive serogroup B meningococcal isolates collected from 2008–2011. This figure shows the number (a) or percentage (b) of isolates with particular combinations of PV expression states. In graph (a), there is further separation based on clonal complex. UA =  unassigned STs. *hmbR only*  =  isolates that possess *hmbR* but not hpuA. *hpuA* only  =  isolates that possess *hpuA* but not *hmbR*. *hmbR:hpuA*  =  isolates that possess both *hmbR* and *hpuA*.

The overall distribution of the combined *hmbR:hpuA* PV statuses and the distribution of *hmbR:hpuA* PV statuses with respect to CC were examined (Figure 5). Excluding isolate i15, 61/79 (77.2%) isolates were genotypically predicted to have at least one system in the ON state. These included approximately 76.9% of isolates among the minor CCs and unassigned STs, and approximately 75.9% of isolates among the major CCs (≥5 isolates) including 85.7% (18/21) of cc41/44, 66.7% (4/6) of cc32, 60% (3/5) of cc213 and 72% (16/22) of cc269 isolates (of which seven were centered on ST-269 and nine were centered on ST-275).

### Phenotypic analyses

In order to correlate the genotypic and phenotypic expression states, the ability to utilise Hb as the sole iron source (indicating the presence of at least one system in the ON configuration) was assessed by growing isolates on iron-restricted media supplemented with different iron sources (figure S3). Excluding isolate i15, phenotyping was indeterminate for 8/79 fully genotyped isolates due to poor growth on MH agar or uncertainty regarding the status of the growth observed (data not shown). Among the remaining 71 isolates, 70 exhibited consistent genotypic/phenotypic PV statuses regarding the expression of at least one Hb receptor. The remaining isolate (*hmbR*-only) was genotypically ON (effective tract length = 9) but phenotypically OFF. Full length sequence analysis of the respective gene (obtained using Illumina sequence technology [Illumina, California, USA]) revealed the presence of a nonsense mutation downstream of the homopolymeric tract, which was consistent with the phenotypic OFF status (data not shown). Full length sequence analysis of *hmbR* and *hpuA* among the remaining isolates was consistent with their respective PV statuses. Genomic sequence data are available on the Pubmlst.org isolate database (http://pubmlst.org/neisseria), corresponding isolate/genome ids are listed in table S1.

**Figure 6:**
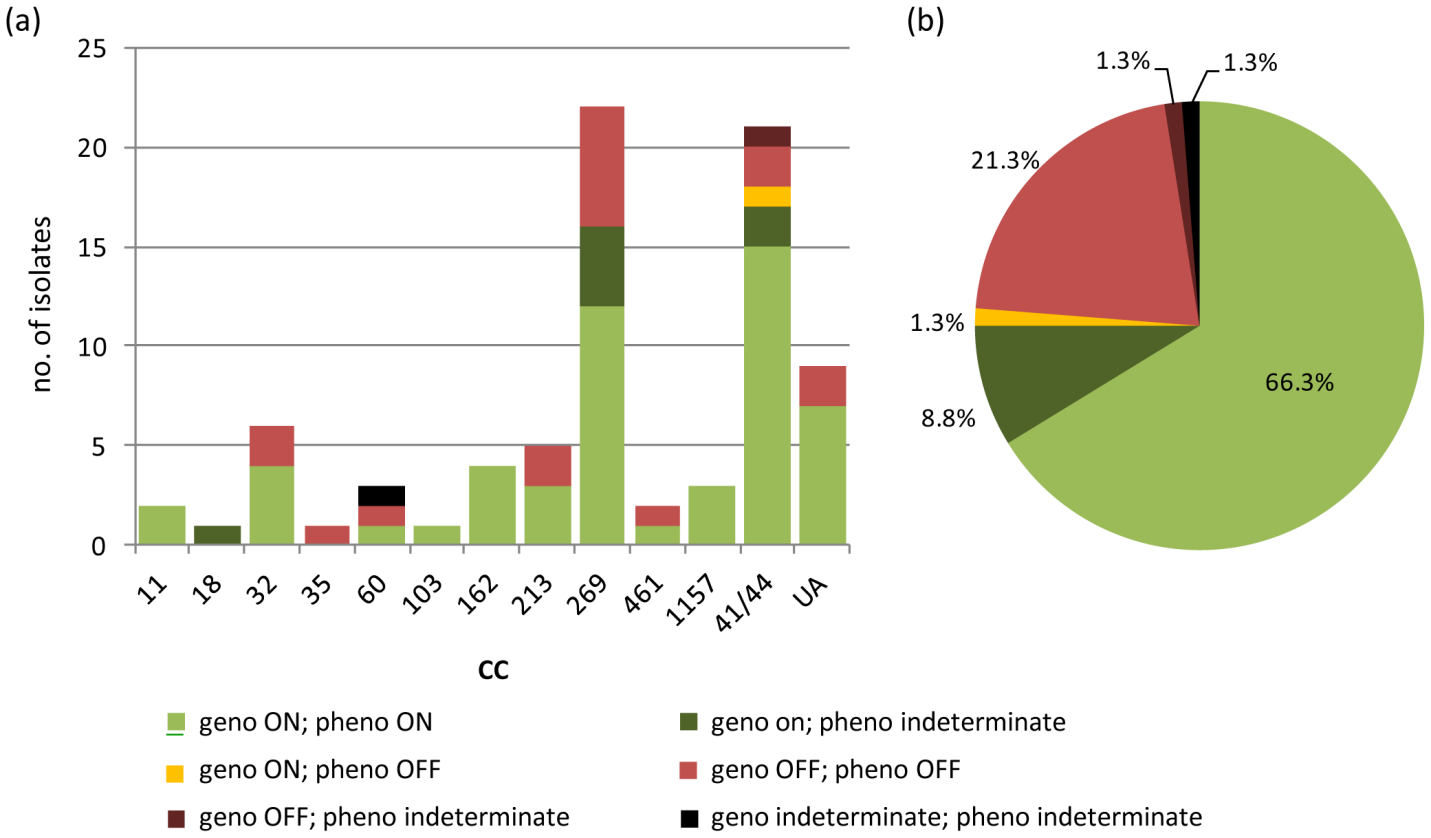
Distribution of the genotypic (phase variable status on/off) and phenotypic expression of at least one of the haemoglobin receptors, HmbR and HpuA, among a representative panel of English, Welsh, and Northern Irish invasive serogroup B meningococcal isolates collected from 2008–2011. geno ON =  at least one receptor gene is in an ON phase variable state. geno OFF =  both receptor genes are in an OFF phase variable state. pheno ON/OFF =  able/unable to grow using haemoglobin as the sole iron source and therefore phenotypically expressing at least one/no haemoglobin receptor/s. geno indeterminate  =  genotypic phase variable state could not be determined owing to ambiguous sequence/fragment analysis. pheno indeterminate  =  phenotypic state could not determined owing to poor growth on MH agar or uncertainty regarding the status of the growth observed (isolate in ON configuration vs relatively large number of ON phase variants against an OFF background). The single isolate with a geno ON pheno OFF status possessed a nonsense mutation downstream of the homopolymeric tract. UA =  unassigned STs.

The overall distribution, and the distribution with respect to CC, of isolates with or without at least one genotypically ON system (with phenotypic support where available) is provided in figure 6. Among all of the isolates, 66.3% (53/80) were confirmed to have at least one receptor in the ON state. A further 8.8% (7/80) were genotypically predicted to be ON but were phenotypically indeterminate. The absence of either receptor in the ON state was confirmed for 22.5% (18/80) of the isolates whilst a further 1.3% (1/80) were genotypically OFF but phenotypically indeterminate. Excluding the indeterminate isolates, these results indicated that 66.7% (12/18) of cc269 isolates, 83.3% (15/18) of cc41/44, 66.7% (4/6) of cc32 isolates and 60% (3/5) of cc213 isolates expressed at least one receptor.

### Comparison of PV status between isolates and corresponding clinical specimens

Clinical specimens with real-time PCR *ctrA* cycle threshold values ≤35 were selected for the study in order to maximise the ability to detect the Hb receptor genes. A nested PCR protocol, consisting of one round of 45 cycles and a second round of 25 cycles, was initially performed using primers spanning the repeat tracts and giving final products of approximately 400–450 (*hmbR*) and 300–350 (*hpuA*) nucleotides. Only 2/76 and 1/34 of the clinical specimens failed to yield *hmbR* and *hpuA* products, respectively (corresponding real-time PCR *ctrA* cycle threshold values ranged from 31.13 to 34.53). A further 69/74 and 19/33 clinical specimens, respectively, yielded 1° fragment peaks corresponding to fragments within 0.41 (mean = 0.12) and 0.42 (mean = 0.29) bases of their respective isolates i.e. corresponding to the same amplicon length. The remaining clinical specimens (for which the corresponding isolates each possessed ≥10 tract repeats) yielded 1° fragment peaks up to 2.25 (mean 1.57) and 1.94 (mean 1.23) bases shorter than those of their respective isolates. All but two of these were brought within range (up to 0.39 bases, mean 0.07) of their respective isolates by eliminating the first round PCR and reducing the total number of cycle repeats to between 25 and 45 cycles. The remaining two specimens did not yield detectable products for the reduced number of PCR cycles. Thus, the PV status was matched for all isolate/specimen pairs for which non-culture PCR products could be obtained using adequately low numbers of PCR cycles.

The *hmbR* and *hpuA* PV status for individual isolates and their corresponding specimens can be viewed alongside phenotypic outcomes in table S1.

## Discussion

Studies have shown that the majority of IMD isolates possess genes for at least one Hb receptor and that the expression of at least one Hb receptor may be important for virulence [16]. This, in conjunction with their surface expression, makes them attractive vaccine candidates. To further evaluate these qualities, studies are required of recent isolates that are epidemiologically representative of specific countries or regions of interest. In addition, the PV status of isolates has to be compared to the prevailing *in vivo* state in order to account for any putative phase variable changes associated with *in vitro* selective pressures occurring during and after initial isolation. These twin objectives were achieved in this study by comparing the distribution and PV status of the Hb receptors in isolates, and their respective specimens, representative of recent IMD in England, Wales and Northern Ireland. Another major outcome of this study was the development of a general approach for studying the contributions of phase variable genes to meningococcal pathogenesis. This is an important contribution as this species contains multiple phase variable genes with key roles in host interactions and in disease progression [18].

Consistent with previous findings [16], [17], *hmbR* (either alone or in conjunction with *hpuA*) was highly represented among these IMD isolates (present in 95%) whilst the presence of *hpuA* as the sole receptor was rare (5%). Interestingly, with the exception of cc461, most of the relatively infrequent CCs (cc11, cc18, cc35, cc60, cc103, cc162 and cc1157) mainly comprised isolates possessing alleles for both receptors. This relatively small collection of isolates exhibited trends consistent with a previous report [16]. The majority of isolates belonging to more prevalent CCs (cc41/44, cc213, cc32 and the ST-269-cluster of cc269) possessed *hmbR* only. This finding highlights the importance of the HmbR protein as a putative virulence factor for the major disease-associated meningococcal strains currently circulating in the UK. A novel finding was that the recently expanding, and broadly antigenically divergent ST-275-cluster of cc269 [19] (constituting approximately half of cc269 isolates in the present study) mainly comprised isolates with both receptors. The increasing prevalence of these isolates could suggest that immunity against the HmbR protein in these isolates has engendered a selection bias for dual Hb receptor strains due to their ability to maintain iron acquisition from Hb while avoiding adaptive immune responses. Longitudinal studies of carriage isolates combined with serology to correlate the PV states of the two receptors with the onset of receptor-specific immunity may serve to shed light on this possibility.

Owing to polymorphisms in or around the homopolymeric repeat tracts, the assertion that tract lengths of 9, 12, 15 and 18 (*hmbR*) or 7, 10, 13, 16 and 19 (*hpuA*) G repeats correspond with the ON expression status [16] did not always hold true for the present isolate panel. Rather, the ‘effective tract length’ between defined up/downstream nucleotides was a more appropriate indicator. With the exception of a single *hmbR*-only isolate with a nonsense mutation downstream of the *hmbR* homopolymeric tract, the predicted PV statuses obtained in this way were supported by the phenotypic status of all isolates that were compatible with the growth media and exhibited non-ambiguous growth status. Western or dot blot analyses using suitable antisera may circumvent the limitations associated with the poor growth of some isolates by enabling direct detection of the respective antigens. These analyses may, however, be susceptible to the influence of low-level phase variants. Immunogold labeling could also be considered since this has the potential to discriminate and quantify such phase variants.

This study also highlighted the need to minimise the number of PCR cycles in order to avoid misleading results arising through strand slippage during amplification. The greater susceptibility to such errors observed among longer homopolymeric tracts and the apparent propensity for contraction of the homopolymeric tract have previously been documented for homopolymeric (poly-T) tracts [22]. As such, the exact tract length for a study isolate with 18+ tract repeats could not be confidently ascertained. Nonetheless, tracts of at least 15 bp in length were successfully quantified using 25 PCR repeat cycles. It should be noted that whilst heterogeneity may have constituted genuine background PV within the respective DNA templates for some isolates or clinical specimens, it is difficult to separate this cause from PCR slippage. Hence, in the present study only the predominant genotype/phenotype was ascertained and considered as representative of a particular strain or specimen.

Despite having undergone a small number of passages on blood containing media during and after initial isolation, the PV statuses of the isolates were highly consistent with those obtained for the corresponding specimens and hence were representative of the disease-associated meningococcal genotype and phenotype. This suggests that the findings of previous studies of these phase variable loci in disease-associated isolates are an accurate reflection of the *in vivo* state [16].

The proportion of isolates genotypically predicted to express at least one Hb receptor (76.3%) was lower than that previously described for IMD isolates (91%) [16]. This is likely to be due to the different distribution of lineages among the respective isolate panels. Tauseef *et al*. [16] utilised 90 IMD strains from the strain panel used to evaluate multilocus sequence typing (MLST) [23]. This includes, for example, three CCs primarily associated with serogroup A (cc1, cc4 and cc5, 29/90, 32.2% overall), none of which were included in the present panel. Similarly, cc269 and cc41/44 constituted 27.5% and 26.3% of the present panel, respectively, but only 1% and 12% of the ‘MLST strain panel’. It is interesting to note, however, that the PV OFF status was not associated with any single lineage, with CCs 32, 35, 60, 213, 461, 41/44 and both cc269 clusters all including ≥24% OFF isolates. Thus the results were not skewed by any single CC.

In light of the relative prevalence of *hmbR* and absence of the ‘*hpuA*-only’ genotype among IMD isolates, it is surprising to note that 19/29 (excluding isolate i15) *hmbR*:*hpuA* isolates possessed *hpuA* in an ON configuration versus only 10/29 for *hmbR*. As speculated for cc269, this may provide further indication of the development of immunity in the UK population against the corresponding HmbR proteins, resulting in a more frequent occurrence of these strains and the expression of HpuAB during IMD. Alternatively, there may be selection for HpuAB during systemic spread of meningococci due to a higher availability of Hb-haptoglobin complexes (the ligand for HpuAB) as opposed to free haemoglobin (the ligand for HmbR). Preferential selection of HpuAB over HmbR has previously been noted during a laboratory acquired meningococcal infection in which the pre-infection organism (*hmbr:hpuA*) was ON for *hmbR* and OFF for *hpuA* [24]. The isolate in question was a so-called mutator, however, the above factors may still have been responsible for the ultimate PV statuses.

Unfortunately, due to the small numbers of specimens, it was not possible to detect whether a particular phase variation state for IMD isolates is required in the CSF as opposed to the blood but this may be an area for more extensive examinations of the roles of these and other phase variable genes.

The current study indicates that ON phase variants occur at a similar frequency in IMD isolates (76%) as previously detected for carriage (71%) isolates [16]. The observed difference between IMD and carriage isolates was less pronounced than that previously detected [16]. Therefore the association of the expression of at least one Hb receptor with IMD may be less robust than initially speculated [16]. However, the carriage isolates previously described were obtained from a localised study of first year university students and included few MenB isolates and multiple isolates arising from clonal expansion [25]. It would be interesting, therefore, to compare the present isolate panel with a comparable, contemporaneous and geographically diverse panel of English, Welsh and Northern Irish MenB carriage isolates to see if the apparent invasive/carriage isolate mismatch still applies. Nonetheless, it seems that expression of at least one Hb receptor may facilitate, but is not essential for, IMD. This is suggestive of broader redundancy of iron uptake mechanisms during IMD, possibly due to the action of Tbp [10].

The predominance of *hmbR*-only isolates among the major CCs and the lack of expression of either *hmbR* or *hpuA* among approximately one fifth of the isolates may reduce the potential coverage of an HmbR/HpuA-containing meningococcal vaccine for MenB in England, Wales and Northern Ireland. Given the necessity of iron for IMD [10], however, a vaccine containing HmbR, HpuA and TbpB as its primary components might circumvent any redundancy in iron uptake mechanisms within the bloodstream. A caveat to this is the prospect that mutants may escape by bypassing the need for the lipoprotein components of the respective systems (HpuA and TbpB) for sufficient iron acquisition [26]; the use of several antigenic components would, nonetheless, reduce the likelihood of vaccine escape. Other MenB vaccines have not focused on potential redundancy-mediated escape in this way [27], [28]. A further benefit to a vaccine incorporating HmbR, HpuA and TbpB is that non-bactericidal antibodies against these receptors may interfere with the process of iron uptake, thereby abrogating virulence [29]. Such effects are not accounted for when measuring serum bactericidal antibody activity which is the correlate of protection for MenB. The vaccine potential of Tbp (either the whole receptor, TbpBA, or the lipoprotein component, TbpB alone) is well documented though the immune responses elicited have been relatively poorly cross-protective, necessitating the use of >1 antigenic variant for broad protection [30]-[33]. Such a need for multiple antigens or antigenic variants has been demonstrated for other subcapsular antigens, including those of the recently licensed 4CMenB vaccine [34] or Pfizer's investigational bivalent recombinant fHbp vaccine [8]. Universal vaccine coverage of MenB is, however, yet to be achieved [9]. Improved coverage for such vaccines might be sought, for example, by incorporating additional or relatively cross-protective antigens/antigenic variants and/or improved adjuvants.

The demonstration of a strong correlation between the PV states of *hmbR* and *hpuA* for IMD isolates and corresponding clinical specimens provides two strategies for determining the *in vivo* states of these phase variable loci during IMD. For specimens with permissive amounts of meningococcal DNA, direct PCR of repeat tracts can be utilised to determine the *in vivo* PV state. For cases where the DNA content of specimens is low, analysis of PV state in a minimally-passaged meningococcal isolate will provide a strong indication of the *in vivo* state. This latter method facilitates studies of phase variable genes as it removes the requirement for ethical approval and because clinical specimens are finite and not always readily available. Both of these methods will indicate the predominant PV state present in the majority of IMD cases. Further studies may be required to determine if meningococcal pathogenesis is associated with a mix of PV states either within or between different anatomical sites. It should also be noted that, in the present study, isolates were cultured on horse blood-containing agar prior to DNA extraction. Alternative media and/or phase variable target genes may affect and/or be affected by relevant selective pressures during culturing that must initially be accounted for (by similar parallel non-culture analyses) on a case by case basis.

Phase variable genes are likely to make significant contributions to IMD and possibly to escape of vaccine-induced immune responses since these genes often encode vaccine antigens (e.g. PorA and NadA), complement resistance factors (e.g. LgtA – one of the enzymes contributing to LPS sialylation), adhesins and invasins (e.g. Opc, Opa and MspA), as well as the haemoglobin-binding proteins described herein. Thus, dissection of the contributions of phase variable genes and other hypermutable loci to IMD and other bacterial diseases is achievable using these methodologies and could provide novel insights into bacterial disease processes.

## Supporting Information

Figure S1 **Workflow of PCR, sequence analyses and fragment analyses performed in the study.** The steps described applied to both hmbR and hpuA unless otherwise stated. aPrimers *hmbR-RF3* and *hmbR-RF4* (for *hmbR*) or *hpuAC* and *hpuA350Rev* (for *hpuA*). For fragment analyses primers *hmbR-RF3* and *hpuA350Rev* were FAM-labelled. bPrimers *hmbRzF* and *hmbRzR* (for *hmbR*) or *hpuAFnest2* and *P26.85* (for *hpuA*). cPrimers *hmbReF* and *hmbRyR* (for *hmbR*) or *Hpu-pmt* and *hpuR-Seq3* (*hpuA*). dPrimers *exl3-seqF* and *exl3-seqR*. ePrimers *hpu-for1* and *hpuArev13*. fNegative reactions (-; failure to amplify products) indicated the absence of the respective gene and prompted confirmatory PCRs using genomic DNA template. gPositive reactions (+; amplification of products) indicated the presence of the gene and prompted sequence analysis of respective PCR product and fragment analysis using a genomic DNA template. M =  size markers. pos =  positive control. neg =  negative control. Gels are depicted schematically.
(PDF)

Figure S2 **Characterisation of homopolymeric tracts and flanking regions.** (a) Chromatogram of the hmbR homopolymeric tract and closely flanking regions for isolate i21. The chromatogram indicates the presence of nine homopolymeric G repeats (black peaks). (b) Fragment analysis for the corresponding FAM-labelled PCR product for isolate i21. The fragment peak (blue) corresponds to a fragment size (S) of 430.22 bases as compared with the flanking GeneScan 500 LIZ size standard fragments (orange peaks; 400 and 450 bases, respectively). (c) Multiple fragments obtained for hmbR for isolate i2 (homopolymeric tract length  = 12 G repeats). Neighbouring peaks represent amplicons differing by a single homopolymeric tract repeat. The primary (1°) peak (corresponding to a fragment length of 433.53 bases) has an area (A) of 1724.2, and the secondary (2°) peak (corresponding to a fragment length of 433.53 bases) has an area of 695.9. The peak area ratio for the 1° and 2° peaks is 2.48 (1724.2/695.9).
(PDF)

Figure S3 **Example of positive and negative results in phenotypic analysis of ability to grow on Hb as the sole iron source.** On Mueller Hinton (MH) agar + desferal (iron chelater), isolate i16 exhibited good growth on haemoglobin (Hb) indicating expression of at least one Hb receptor. Isolate i106 did not grow using Hb as the sole iron source indicating that no Hb receptor was expressed. Controls: both isolates grew on transferrin (Tf) as a sole iron source and on untreated MH agar. Neither isolate grew on H20 supplement on iron depleted MH agar.
(PDF)

Table S1 **Genotypic and phenotypic phase variation status of** ***hmbR*** **and** ***hpuA*** **among a representative panel of English, Welsh and Northern Irish invasive serogroup B meningococcal isolates and their corresponding clinical specimens.** ^a^Presence of at least one gene in the ON configuration (ON =  at least one gene present in ON configuration; OFF =  no genes in ON configuration). ^b^Ability to utilise Hb as sole iron source. ^c^Isolate contained >18 homopolymeric tract repeats. ^d^Poor growth on Mueller Hinton agar or uncertainty regarding growth status. ^e^Specimen did not yield detectable PCR products at reduced number of PCR cycles. ^f^Indicates whether ST is centred upon ST-269 (269) or ST-275 (275) by eBURST analysis. ^g^isolate/genome id on the pubmlst database (http://pubmlst.org/perl/bigsdb/bigsdb.pl?db=pubmlst_neisseria_isolates). *n/a = * not applicable (*hmbR^-^/hpuA^-^*) same  =  same as that of corresponding isolate.
(PDF)
