# Supplementary material for: The Distribution and ‘In Vivo’ Phase Variation Status of Haemoglobin Receptors in Invasive Meningococcal Serogroup B Disease: Genotypic and Phenotypic Analysis
Source: PLoS One. 2013 Sep 30;8(9):e76932. doi: 10.1371/journal.pone.0076932 (PMC3786947; doi:10.1371/journal.pone.0076932)
Supplement: Figure S1 — Workflow of PCR, sequence analyses and fragment analyses performed in the study. The steps described applied to both hmbR and hpuA unless otherwise stated. aPrimers hmbR-RF3 and hmbR-RF4 (for hmbR) or hpuAC and hpuA350Rev (for hpuA). For fragment analyses primers hmbR-RF3 and hpuA350Rev were FAM-labelled. bPrimers hmbRzF and hmbRzR (for hmbR) or hpuAFnest2 and P26.85 (for hpuA). cPrimers hmbReF and hmbRyR (for hmbR) or Hpu-pmt and hpuR-Seq3 (hpuA). dPrimers exl3-seqF and exl3-seqR. ePrimers hpu-for1 and hpuArev13. fNegative reactions (-; failure to amplify products) indicated the absence of the respective gene and prompted confirmatory PCRs using genomic DNA template. gPositive reactions (+; amplification of products) indicated the presence of the gene and prompted sequence analysis of respective PCR product and fragment analysis using a genomic DNA template. M = size markers. pos = positive control. neg = negative control. Gels are depicted schematically. (PDF) [file pone.0076932.s001.pdf]

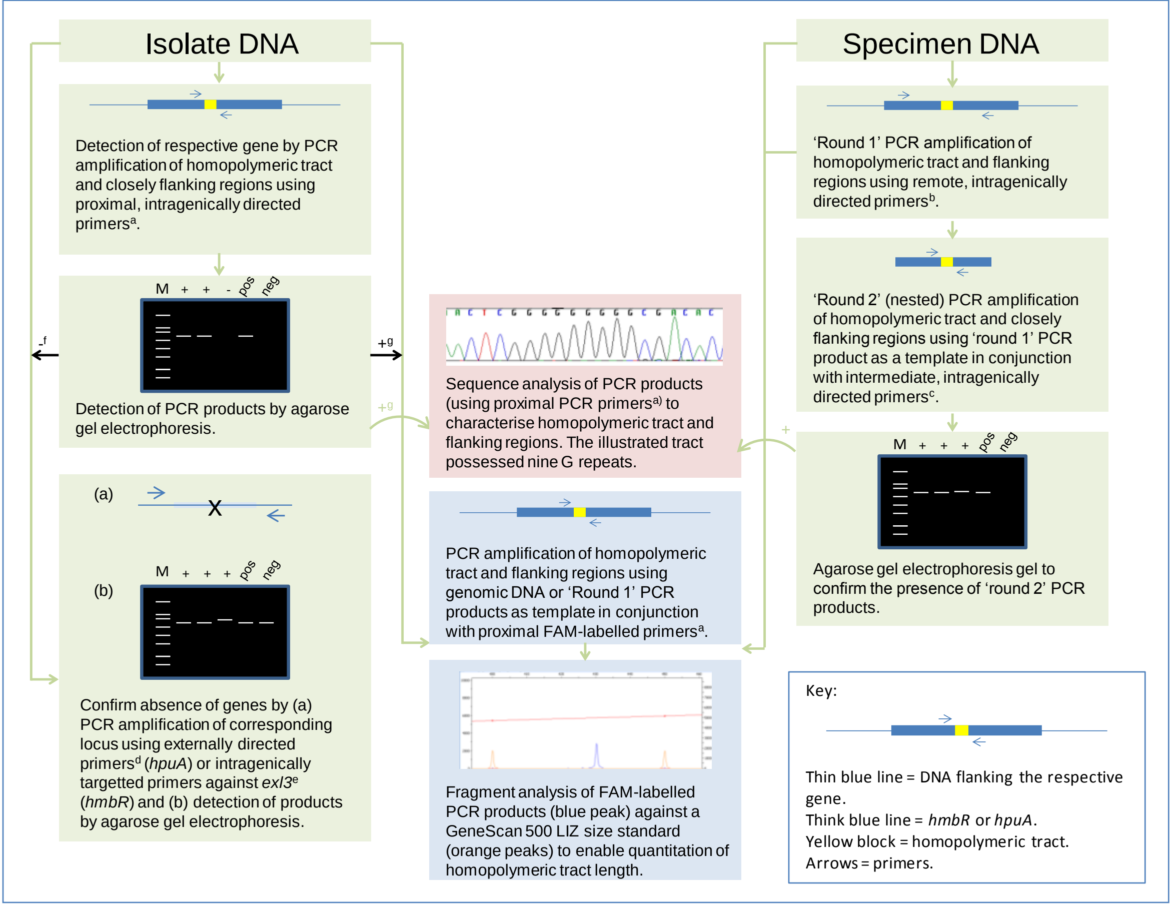
Isolate DNA
Detection of respective gene by PCR amplification of homopolymeric tract and closely flanking regions using proximal, intragenically directed primers[a].
M + + - pos neg
Detection of PCR products by agarose gel electrophoresis.
-[f]
+[g]
+[g]
(a)
X
(b)
M + + + pos neg
Confirm absence of genes by (a) PCR amplification of corresponding locus using externally directed primers[d] (*hpuA*) or intragenically targetted primers against *exl3*[e] (*hmbR*) and (b) detection of products by agarose gel electrophoresis.
Sequence analysis of PCR products (using proximal PCR primers[a)] to characterise homopolymeric tract and flanking regions. The illustrated tract possessed nine G repeats.
PCR amplification of homopolymeric tract and flanking regions using genomic DNA or 'Round 1' PCR products as template in conjunction with proximal FAM-labelled primers[a].
Fragment analysis of FAM-labelled PCR products (blue peak) against a GeneScan 500 LIZ size standard (orange peaks) to enable quantitation of homopolymeric tract length.
Specimen DNA
'Round 1' PCR amplification of homopolymeric tract and flanking regions using remote, intragenically directed primers[b].
'Round 2' (nested) PCR amplification of homopolymeric tract and closely flanking regions using 'round 1' PCR product as a template in conjunction with intermediate, intragenically directed primers[c].
+
M + + + pos neg
Agarose gel electrophoresis gel to confirm the presence of 'round 2' PCR products.
Key:
Thin blue line = DNA flanking the respective gene.
Think blue line = *hmbR* or *hpuA*.
Yellow block = homopolymeric tract.
Arrows = primers.
