# Supplementary figures and images for: The Distribution and ‘In Vivo’ Phase Variation Status of Haemoglobin Receptors in Invasive Meningococcal Serogroup B Disease: Genotypic and Phenotypic Analysis
Source: PLoS One. 2013 Sep 30;8(9):e76932. doi: 10.1371/journal.pone.0076932 (PMC3786947; doi:10.1371/journal.pone.0076932)

(a)

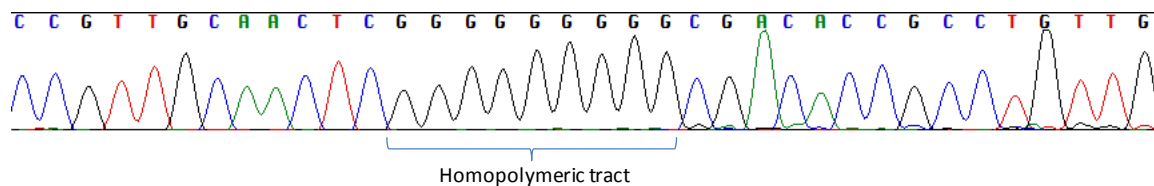

(b)

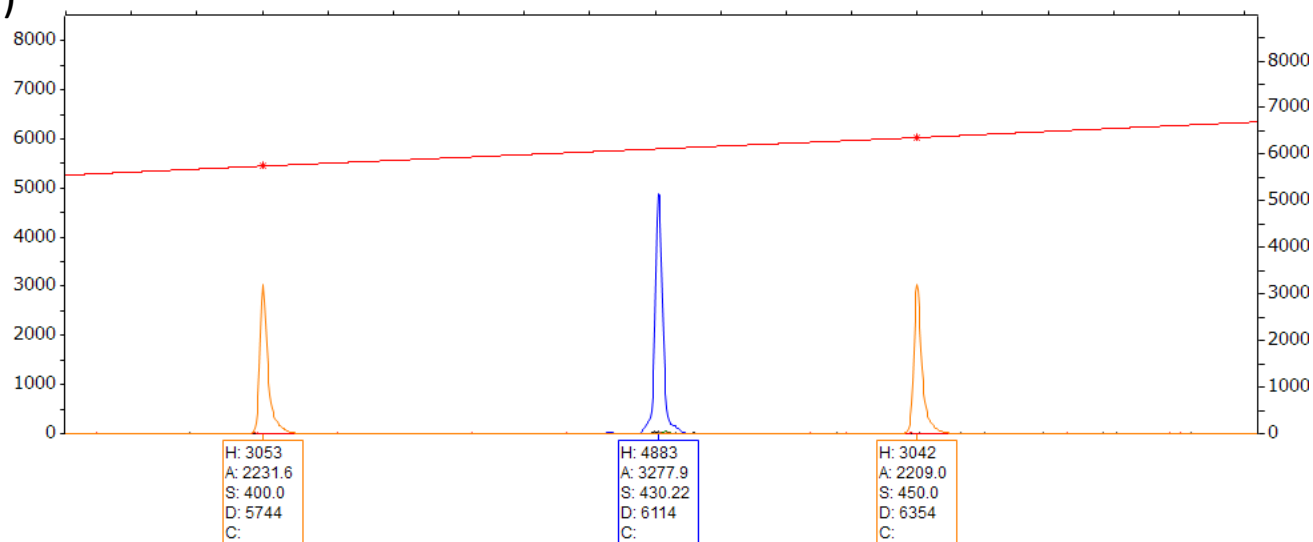

(c)

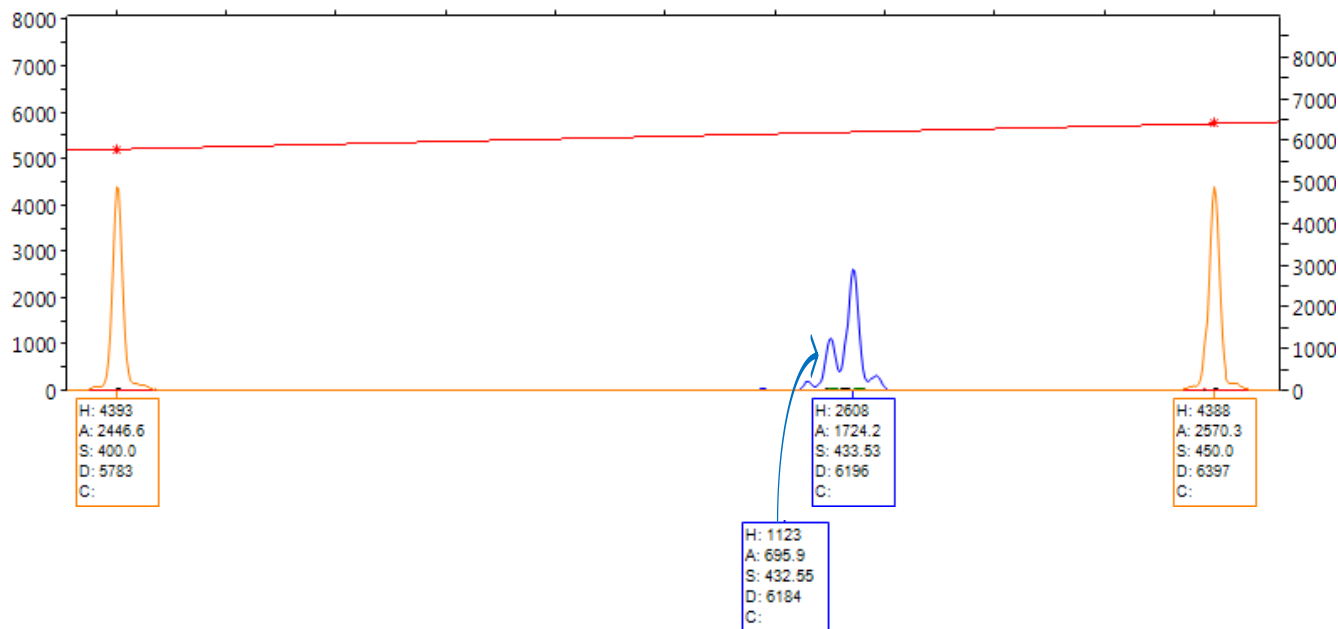

Supplement: Figure S2 — Characterisation of homopolymeric tracts and flanking regions. (a) Chromatogram of the hmbR homopolymeric tract and closely flanking regions for isolate i21. The chromatogram indicates the presence of nine homopolymeric G repeats (black peaks). (b) Fragment analysis for the corresponding FAM-labelled PCR product for isolate i21. The fragment peak (blue) corresponds to a fragment size (S) of 430.22 bases as compared with the flanking GeneScan 500 LIZ size standard fragments (orange peaks; 400 and 450 bases, respectively). (c) Multiple fragments obtained for hmbR for isolate i2 (homopolymeric tract length = 12 G repeats). Neighbouring peaks represent amplicons differing by a single homopolymeric tract repeat. The primary (1°) peak (corresponding to a fragment length of 433.53 bases) has an area (A) of 1724.2, and the secondary (2°) peak (corresponding to a fragment length of 433.53 bases) has an area of 695.9. The peak area ratio for the 1° and 2° peaks is 2.48 (1724.2/695.9). (PDF) [file pone.0076932.s002.pdf]

## MH agar

## MH agar + desferal

supplement

Hb

Tf

H<sub>2</sub>O

i10

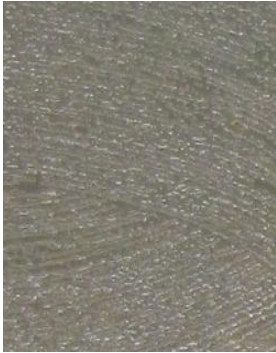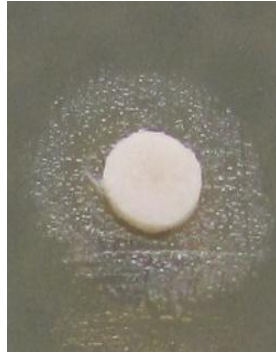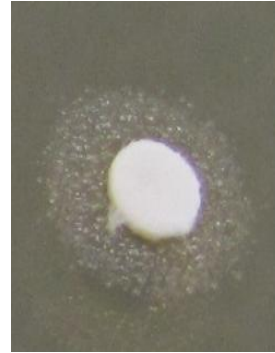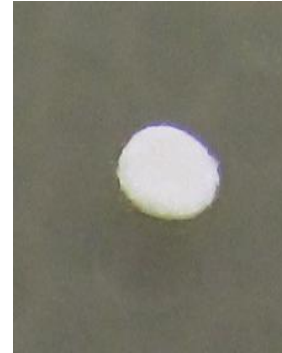

i106

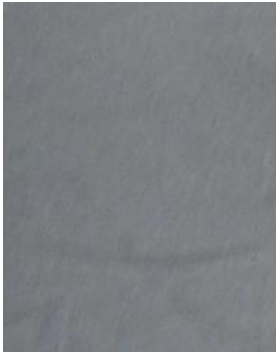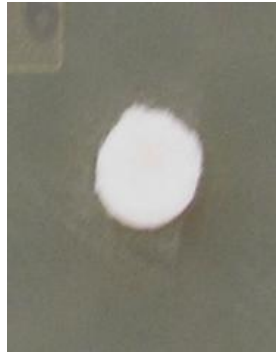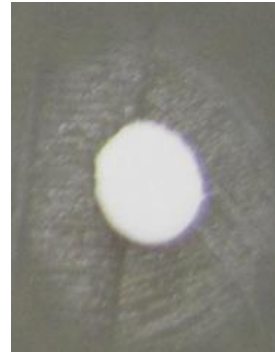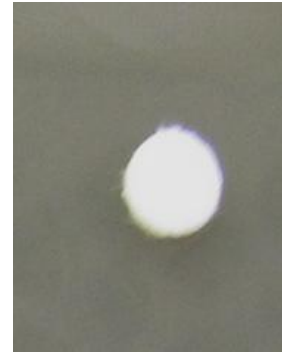

isolate

Supplement: Figure S3 — Example of positive and negative results in phenotypic analysis of ability to grow on Hb as the sole iron source. On Mueller Hinton (MH) agar + desferal (iron chelater), isolate i16 exhibited good growth on haemoglobin (Hb) indicating expression of at least one Hb receptor. Isolate i106 did not grow using Hb as the sole iron source indicating that no Hb receptor was expressed. Controls: both isolates grew on transferrin (Tf) as a sole iron source and on untreated MH agar. Neither isolate grew on H20 supplement on iron depleted MH agar. (PDF) [file pone.0076932.s003.pdf]
