# Supplementary material for: The Distribution and ‘In Vivo’ Phase Variation Status of Haemoglobin Receptors in Invasive Meningococcal Serogroup B Disease: Genotypic and Phenotypic Analysis
Source: PLoS One. 2013 Sep 30;8(9):e76932. doi: 10.1371/journal.pone.0076932 (PMC3786947; doi:10.1371/journal.pone.0076932)
Supplement: Table S1 — Genotypic and phenotypic phase variation status of hmbR and hpuA among a representative panel of English, Welsh and Northern Irish invasive serogroup B meningococcal isolates and their corresponding clinical specimens. aPresence of at least one gene in the ON configuration (ON = at least one gene present in ON configuration; OFF = no genes in ON configuration). bAbility to utilise Hb as sole iron source. cIsolate contained >18 homopolymeric tract repeats. dPoor growth on Mueller Hinton agar or uncertainty regarding growth status. eSpecimen did not yield detectable PCR products at reduced number of PCR cycles. fIndicates whether ST is centred upon ST-269 (269) or ST-275 (275) by eBURST analysis. gisolate/genome id on the pubmlst database (http://pubmlst.org/perl/bigsdb/bigsdb.pl?db=pubmlst_neisseria_isolates). n/a = not applicable (hmbR-/hpuA-) same = same as that of corresponding isolate. (PDF) [file pone.0076932.s004.pdf]

Table S1. Genotypic and phenotypic phase variation status of *hmbR* and *hpuA* among a representative panel of English, Welsh and Northern Irish invasive serogroup B meningococcal isolates and their corresponding clinical specimens.

Isolate/ specimen	Isolate/ genome id[g]	Sequence type	Clonal complex (cluster[f])	Specimen	PV status					
					Isolates				Specimens (genotypic, versus corresponding isolate)	
					Genotypic (effective homopolymeric tract length/absolute tract length where different)			Phenotypic[b]		
					hmbR	*hpuA*	Overall[a]		*hmbR*	*hpuA*
i1/s1	20066	11	11	Blood	ON (9)	ON (10)	on	on	same	same
i2/s2	20215	11	11	CSF	ON (12)	ON (10)	on	on	same	same
i28/s28	20765	9328	18	Blood	ON (9)	OFF (6)	on	indeterminate[d]	same	same
i5/s5	20297	32	32	Blood	OFF (10)	ON (13)	on	on	same	same
i88/s88	20740	32	32	Plasma	ON (15)	*n/a*	on	on	same	*n/a*
i89/s89	20747	33	32	Blood	ON (9)	*n/a*	on	on	same	*n/a*
i6/s6	20139	259	32	Blood	ON (12)	*n/a*	on	on	same	*n/a*
i7/s7	20772	749	32	Blood	OFF (10)	*n/a*	off	off	same	*n/a*
i4/s4	20789	2503	32	Plasma	OFF (11/9)	*n/a*	off	off	same	*n/a*
i90/s90	20739	35	35	Blood	OFF (8)	ON (12/13)	off	off	same	same
i15/s15	20790	60	60	Blood	OFF (10)	indeterminate	Indeterminate[c]	Indeterminate[d]	same	same
i91/s91	20741	7788	60	Blood	OFF (7)	OFF (11/12)	off	off	same	same
i19/s19	20287	9327	60	Blood	OFF (8)	ON (13/14)	on	on	same	same
i13/s13	20248	9239	103	Blood	OFF (11)	ON (10)	on	on	same	same
i583/s62	20804	162	162	Blood	OFF (8)	ON (10)	on	on	same	same
i22/s22	20794	162	162	Blood	OFF (8)	ON (13)	on	on	same	same
i63/s63	20756	162	162	Blood	OFF (8)	ON (13)	on	on	same	same
i21/s21	20786	8383	162	Blood	ON (9)	ON (10)	on	on	same	same
i23/s23	20797	213	213	Blood	OFF (7)	*n/a*	off	off	same	*n/a*
i26/s26	20759	213	213	Blood	ON (9)	*n/a*	on	on	same	*n/a*

Isolate/ specimen	Isolate/ genome id[g]	Sequence type	Clonal complex (cluster[f])	Specimen	PV status					
					Isolates				Specimens (genotypic, versus corresponding isolate)	
					Genotypic (effective homopolymeric tract length/absolute tract length where different)			Phenotypic[b]	*hmbR*	*hpuA*
					hmbR	*hpuA*	Overall[a]			
i29/s29	20769	213	213	Blood	OFF (10)	*n/a*	off	off	same	*n/a*
i31/s31	20771	213	213	Blood	ON (9)	*n/a*	on	on	same	*n/a*
i27/s27	20763	2388	213	Serum	ON (9)	*n/a*	on	on	same	*n/a*
i48/s48	20793	461	461	Blood	OFF (13)	*n/a*	off	off	same	*n/a*
i55/s55	20753	461	461	Blood	ON (15)	*n/a*	on	on	indeterminate[e]	*n/a*
i60/s60	20780	1157	1157	Blood	*n/a*	ON (10/11)	on	on	*n/a*	same
i61/s61	20035	1157	1157	CSF	ON (9)	ON (13/14)	on	on	same	same
i59/s59	20777	3149	1157	CSF	ON (9)	OFF (9/10)	on	on	same	same
i9/s9	20775	13	269 (269)	Blood	OFF (11)	OFF (9)	off	off	same	same
i32/s32	20808	269	269 (269)	Blood	ON (9)	*n/a*	on	indeterminate[d]	same	*n/a*
i35/s35	20752	269	269 (269)	Blood	ON (9)	*n/a*	on	indeterminate[d]	same	*n/a*
i39/s39	20807	269	269 (269)	Blood	ON (9)	*n/a*	on	on	same	*n/a*
i40/s40	20774	269	269 (269)	Blood	ON (9)	*n/a*	on	Indeterminate[d]	same	*n/a*
i41/s41	20778	269	269 (269)	Blood	OFF (10)	*n/a*	off	off	same	*n/a*
i42/s42	20767	269	269 (269)	CSF	OFF (8)	*n/a*	off	off	same	*n/a*
i96/s96	20744	269	269 (269)	CSF	OFF (8)	*n/a*	off	off	same	*n/a*
i18/s18	20119	479	269 (269)	Blood	ON (9)	*n/a*	on	on	same	*n/a*
i37/s37	20806	1214	269 (269)	Blood	ON (9)	*n/a*	on	on	same	*n/a*
i36/s36	20754	9240	269 (269)	Blood	ON (9)	*n/a*	on	indeterminate[d]	no product	*n/a*
i92/s92	20784	275	269 (275)	Blood	ON (9)	OFF (9)	on	on	same	same
i95/s95	20743	275	269 (275)	Blood	*n/a*	ON (10)	on	on	*n/a*	same
i97/s97	20745	275	269 (275)	Blood	OFF (8)	ON (10)	on	on	same	same

Isolate/ specimen	Isolate/ genome id[g]	Sequence type	Clonal complex (cluster[f])	Specimen	PV status					
					Isolates				Specimens (genotypic, versus corresponding isolate)	
					Genotypic (effective homopolymeric tract length/absolute tract length where different)			Phenotypic[b]	*hmbR*	*hpuA*
					hmbR	*hpuA*	Overall[a]			
i45/s45	20779	1161	269 (275)	Blood	ON (9)	ON (4)	on	on	same	same
i46/s46	20783	1161	269 (275)	Blood	OFF (10)	OFF (9)	off	off	no product	no product
i47/s47	20787	1161	269 (275)	Blood	OFF (8)	ON (10)	on	on	same	same
i50/s50	20796	1161	269 (275)	Blood	OFF (10)	ON (13)	on	on	same	same
i98/s98	20737	1161	269 (275)	Blood	OFF (10)	ON (13)	on	on	same	same
i11/s11	19986	1163	269 (275)	Blood	ON (9)	ON (10/9)	on	on	same	same
i53/s53	20751	1991	269 (275)	Blood	ON (9)	OFF (8)	on	on	same	same
i52/s52	20750	9242	269 (275)	Blood	OFF (10)	OFF (9)	off	off	same	same
i105/s105	20742	41	41/44	Serum	ON (9)	*n/a*	on	on	same	*n/a*
i106/s106	20748	41	41/44	CSF	OFF (8)	*n/a*	off	off	same	*n/a*
i14/s14	20788	41	41/44	Blood	ON (9)	*n/a*	on	on	same	*n/a*
i30/s30	20770	41	41/44	CSF	ON (9)	*n/a*	on	on	same	*n/a*
i70/s70	20781	41	41/44	Blood	OFF (8)	ON (13)	on	on	same	same
i81/s81	20755	41	41/44	Blood	OFF (8)	*n/a*	off	Indeterminate[d]	same	*n/a*
i85/s85	20762	41	41/44	Blood	ON (9)	*n/a*	on	Indeterminate[d]	same	*n/a*
i75/s75	20798	154	41/44	Blood	ON (9)	*n/a*	on	on	same	*n/a*
i74/s74	20795	303	41/44	Blood	ON (9)	*n/a*	on	on	same	*n/a*
i25/s25	20758	318	41/44	Blood	ON (9)	*n/a*	on	on	same	*n/a*
i77/s77	20802	340	41/44	Blood	ON (9)	*n/a*	on	on	same	*n/a*
i86/s86	20764	340	41/44	Blood	ON (9)	*n/a*	on	on	same	*n/a*
i16/s16	20766	839	41/44	Blood	ON (9)	*n/a*	on	on	same	*n/a*
i101/s101	20738	1194	41/44	Blood	ON (9)	*n/a*	on	on	same	*n/a*

Isolate/ specimen	Isolate/ genome id[g]	Sequence type	Clonal complex (cluster[f])	Specimen	PV status					
					Isolates				Specimens (genotypic, versus corresponding isolate)	
					Genotypic (effective homopolymeric tract length/absolute tract length where different)			Phenotypic[b]		
					hmbR	*hpuA*	Overall[a]		*hmbR*	*hpuA*
i69/s69	20809	1788	41/44	Blood	ON (6)	*n/a*	on	Indeterminate[d]	same	*n/a*
i73/s73	20792	3754	41/44	Blood	ON (9)	*n/a*	on	on	same	*n/a*
i82/s82	20757	4100	41/44	Blood	ON (9)	*n/a*	on	off	same	*n/a*
i80/s80	20805	6761	41/44	Blood	ON (9)	*n/a*	on	on	same	*n/a*
i76/s76	20800	7385	41/44	Blood	ON (9)	*n/a*	on	on	same	*n/a*
i83/s83	20760	9160	41/44	Blood	OFF (8)	*n/a*	off	off	same	*n/a*
i8/s8	20773	9238	41/44	Blood	ON (9)	*n/a*	on	on	same	*n/a*
i49/s49	20791	1159	UA	CSF	*n/a*	ON (10)	on	on	*n/a*	same
i34/s34	19965	1575	UA	Blood	OFF (10)	ON (7)	on	on	same	same
i107/s107	20746	1867	UA	Blood	ON (12)	*n/a*	on	on	same	*n/a*
i3/s3	20767	3280	UA	CSF	ON (9)	*n/a*	on	on	same	*n/a*
i17/s17	20118	4051	UA	Blood	*n/a*	OFF (8/9)	off	off	*n/a*	same
i10/s10	20776	5151	UA	CSF	ON (9)	*n/a*	on	on	same	*n/a*
i87/s87	20801	5335	UA	Blood	OFF (10)	OFF (12)	off	off	same	same
i20/s20	20331	8944	UA	Blood	ON (9)	*n/a*	on	on	same	*n/a*
i51/s51	20799	9241	UA	CSF	OFF (8)	ON (13)	on	on	same	indeterminate[e]

[a]Presence of at least one gene in the ON configuration (ON = at least one gene present in ON configuration; OFF = no genes in ON configuration).
[b]Ability to utilise Hb as sole iron source.
[c]Isolate contained >18 homopolymeric tract repeats.
[d]Poor growth on Mueller Hinton agar or uncertainty regarding growth status.
[e]Specimen did not yield detectable PCR products at reduced number of PCR cycles.
[f]Indicates whether ST is centred upon ST-269 (269) or ST-275 (275) by eBURST analysis.
[g]isolate/genome id on the pubmlst database (http://pubmlst.org/perl/bigsdb/bigsdb.pl?db=pubmlst_neisseria_isolates).
n/a = not applicable (*hmbR*[-]/*hpuA*[-])
same = same as that of corresponding isolate.
